# A coiled conformation of amyloid-β recognized by antibody C706

**DOI:** 10.1186/s13195-017-0296-0

**Published:** 2017-08-22

**Authors:** Alexey Teplyakov, Galina Obmolova, Gary L. Gilliland

**Affiliations:** Janssen Research and Development, LLC, 1400 McKean Road, Spring House, PA 19477 USA

**Keywords:** Alzheimer’s disease, β-Amyloid, Antibody, Crystal structure, Epitope, Immunization

## Abstract

**Background:**

β-Amyloid (Aβ) peptide is believed to play a pivotal role in the development of Alzheimer’s disease. Passive immunization with anti-Aβ monoclonal antibodies may facilitate the clearance of Aβ in the brain and may thus prevent the downstream pathology. Antibodies targeting the immunodominant N-terminal epitope of Aβ and capable of binding both the plaques and soluble species have been most efficacious in animal models. Structural studies of such antibodies with bound Aβ peptides provided the basis for understanding the mechanisms of action and the differences in potency. To gain further insight into the structural determinants of antigen recognition and the preferential Aβ conformations, we determined the crystal structure of murine antibody C706 in complex with the N-terminal Aβ 1–16 peptide sequence.

**Methods:**

The antigen-binding fragment of C706 was expressed in HEK293 cells and was crystallized in complex with the Aβ peptide. The X-ray structure was determined at 1.9-Å resolution.

**Results:**

The binding epitope of C706 is centered on residues Arg5 and His6, which provide the majority of interactions. Unlike most antibodies, C706 recognizes a coiled rather than extended conformation of Aβ.

**Conclusions:**

Comparison with other antibodies targeting the N-terminal section of Aβ suggests that the conformation of the bound peptide may be linked to the immunization protocol and may reflect the preference for the extended conformation in the context of a longer Aβ peptide as opposed to the coiled conformation in the isolated short peptide.

## Background

Alzheimer’s disease (AD), a progressive neurodegenerative disease, is characterized by hyperphosphorylation of the microtubule-associated protein tau in neurons and by extracellular deposits of β-amyloid (Aβ) plaques in the brain [1]. Aβ plaque formation, which plays a central role in AD pathogenesis, is promoted by elevated levels of the self-aggregating 42-amino acid peptide (Aβ_42_) of the amyloid precursor protein (APP). The normal function of APP or its proteolytic products is unknown.

Several immunological approaches directed toward interrupting the amyloid cascade [2] are currently under investigation [3–5]. One approach that targets amyloid plaque clearance employs the peripheral administration of Aβ-specific monoclonal antibodies (mAbs) [6, 7]. In this approach, antibodies bind circulating soluble Aβ, changing the Aβ concentrations between the central nervous system and plasma. According to the peripheral sink model, the gradient in Aβ concentration promotes its export from the brain and dissolution of plaques. Passive immunization with anti-Aβ antibodies demonstrated activity in transgenic animal models [6, 7] and is being evaluated in clinical trials [8].

Anti-Aβ mAbs considered as potential therapeutics differ in their mechanisms of action and binding epitopes. Those targeting the N-terminal linear epitope of Aβ are capable of binding both the plaques and soluble species and have been most efficacious [9]. The N-terminal region of Aβ constitutes the immunodominant B-cell epitope of Aβ [10] and lacks T-cell epitopes implicated in the toxicity upon active immunization with fibrillar Aβ [11]. This epitope is therefore a leading target for the development of anti-Aβ immunotherapies [12].

mAb C706 was raised in mice immunized with the N-terminal DAEFRHD sequence of human Aβ [13]. It binds Aβ_42_ with a dissociation constant of 13 nM and effectively inhibits Aβ_42_ oligomer-induced toxicity in rat PC-12 cells [14]. To gain insight into molecular interactions and the mechanism of action of C706, we have determined the crystal structure of the C706 antigen-binding fragment (Fab) in complex with Aβ_16_. Comparison with other mAbs that recognize the same epitope revealed two distinct conformations adopted by the N-terminal portion of Aβ, indicating the specificity of each mAb toward a particular fraction of the Aβ pool.

## Methods

### Materials

A chimeric Fab fragment of mAb C706 was constructed by fusing the murine variable domains with human immunoglobulin G1/κ constant domains. The Fab was expressed in HEK293 cells (Thermo Fisher Scientific, Waltham, MA, USA) using Lonza (Walkersville, MD, USA)-based vectors and was purified by cation exchange and size exclusion chromatography using, respectively, Mono S and Superdex 200 columns (GE Healthcare Bio-Sciences, Pittsburgh, PA, USA). The Aβ 1–16 peptide sequence (Aβ_16_) was synthesized with an acetylated N-terminus and an amidated C-terminus. The amino acid sequence of the peptide is Act-DAEFRHDSGYEVHHQK-NH_2_.

### Crystallization

The lyophilized Aβ_16_ was reconstituted in 20 mM Tris buffer, pH 8.5. The Fab-Aβ_16_ complex was prepared by mixing 3 mg of Fab with 0.6 mg of Aβ_16_ at a molar ratio of 1:5 (excess of peptide). The mixture was incubated for 20 minutes, concentrated to 16 mg/ml, and used for crystallization.

Crystallization of the complex was carried out by the vapor diffusion method at 20 °C using an Oryx 4 robot (Douglas Instruments, Hungerford, UK). The initial screening was performed with the PEG/Ion HT crystallization screen (Hampton Research, Aliso Viejo, CA, USA). Crystals suitable for X-ray analysis were obtained by microseed matrix screening [15] from 2.0 M ammonium sulfate in 0.1 M acetate buffer, pH 4.5.

### X-ray data collection and structure determination

For X-ray data collection, one Fab-Aβ_16_ crystal was soaked for a few seconds in the mother liquor supplemented with 30% glycerol and flash frozen in the stream of nitrogen at 100 K. X-ray diffraction data were collected using a MicroMax-007HF microfocus X-ray generator equipped with an Osmic VariMax confocal optics, a Saturn 944 detector, and an X-stream 2000 cryocooling system (Rigaku, The Woodlands, TX, USA). Diffraction intensities were detected over 650 degrees of crystal rotation with the exposure time of 2 minutes per half-degree image to the maximum resolution of 1.9 Å. The X-ray data were processed with the *XDS* program [16]. X-ray data are given in Table 1.

**Table 1:** Crystal data, X-ray data, and refinement statistics

Crystal data type	Statistics
Space group	P2_1_2_1_2_1_
Unit cell axes, Å	65.20, 69.88, 104.86
Molecules per asymmetric unit	1
V_m_ (Å^3^/Da)/solvent content, %	2.47/50
X-ray data	
Resolution, Å	30–1.9 (2.0–1.9)
Number of measured reflections	591,216 (9830)
Number of unique reflections	34,530 (1542)
Completeness, %	92.3 (56.7)
Redundancy	17.1 (6.4)
R_sym_(I)	0.068 (0.188)
Mean I/σ(I)	36.5 (9.7)
B factor from Wilson plot, Å^2^	22.3
Refinement	
Resolution, Å	20.0–1.9
R_cryst_	0.198
R_free_	0.236
Number of all atoms	3714
Number of water molecules	331
Bond lengths RMSD, Å	0.007
Bond angles RMSD, degrees	1.2
Mean B factor from model, Å^2^	20.8
Ramachandran plot, most favored, %	93.2
Ramachandran plot, disallowed, %	0.3

*RMSD* Root-mean-square deviation

Numbers in parentheses refer to the highest-resolution shell

The structure was determined by molecular replacement with the *Phaser* program [17] using the C706 Fab structure (3mcl; [13]) as a search model. When the Fab was positioned in the unit cell, the Aβ peptide was manually traced in the electron density using *Coot* [18]. The structure was refined with *Refmac5* [19]. Refinement statistics are given in Table 1. All crystallographic calculations were performed with the *CCP4* suite of programs [20]. Ramachandran statistics were calculated with *PROCHECK* [21]. Figures were prepared with PyMol (Schrödinger, Cambridge, MA, USA). The Chothia numbering scheme of antibody residues [22] is used throughout this article.

## Results

**Fig. 1:**
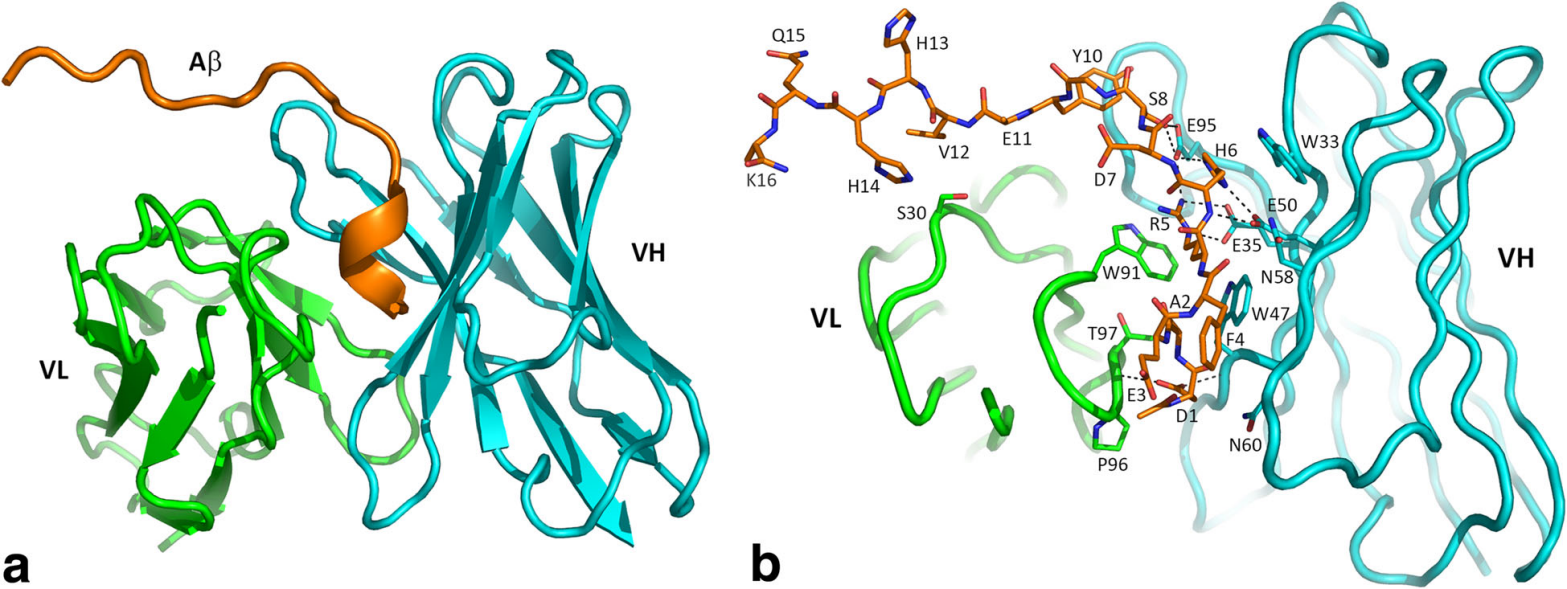
Interactions between C706 and the β-amyloid 1–16 peptide sequence (Aβ_16_). **a** Cartoon diagram of Aβ_16_ bound to the C706 antigen-binding fragment (Fab). **b** Aβ_16_ and C706 paratope residues represented as sticks. Side chains of Glu11 and Lys16 were not included in the model. *Green* = variable domain of the light chain (VL), *cyan* = variable domain of the heavy chain (VH), and *orange* = Aβ_16_. Hydrogen bonds are shown as *dashed lines*

The structure of the C706 Fab-Aβ_16_ complex was determined at 1.9-Å resolution. All 16 residues of Aβ_16_ and all complementarity-determining region (CDR) residues are clearly defined in the electron density. The CDRs in C706 are relatively short, particularly in the light chain. CDR L1 and CDR L3 contain, respectively, ten and eight residues, which is one residue shorter than their typical lengths [23]. CDR H3 is in an open conformation leaning toward CDR L2. As a result, the binding surface exhibits a pronounced crevice between CDRs H1 and H2 on one side and CDRs H3, L1, and L3 on the other side. Aβ_16_ is bound in this groove with its N-terminus close to the N-terminus of the variable domain of the light chain (VL) (Fig. 1). The surface area of the Fab buried upon binding of Aβ_16_ is about 600 Å^2^, which is a typical value for linear epitopes [24]. The N-terminal half of Aβ_16_ makes numerous contacts with C706, whereas the C-terminal half has very few contacts. Only Tyr10 and Val12 are within van der Waals distance from CDRs H3 and L1, respectively. Nevertheless, residues 9–16 are not disordered, probably owing to the contacts with a symmetry-related Fab molecule.

Residues 1–5 of Aβ_16_ adopt a coiled conformation. Three residues, Asp1, Arg5, and His6, provide almost all antibody-antigen interactions. The side chains of Arg5 and His6 form a stack with flanking Trp91(L) and Trp33(H). They also form a number of hydrogen bonds with Glu35, Glu50, and Glu95 at the bottom of the binding pocket (Fig. 1). The carboxyl group of Asp1 makes H-bonds to the main-chain amino groups of Trp47(H) and Thr97(L), thus bridging the variable domain of the heavy chain (VH) and VL. The acetyl group at the N-terminus of the Aβ_16_ peptide makes no contacts with the antibody and probably has no effect on the binding of the peptide. Residues Glu3, Phe4, and Asp7 point away from the antibody. Ser8 makes an H-bond with Glu95 of CDR H3. Whereas the side chains of several Aβ residues provide key interactions, the main-chain carbonyl and amino groups are not in direct contact with the antibody. Seven water molecules and two sulfate ions bridge the Aβ backbone and the CDR residues through H-bonds, thus complementing the interaction.

**Fig. 2:**
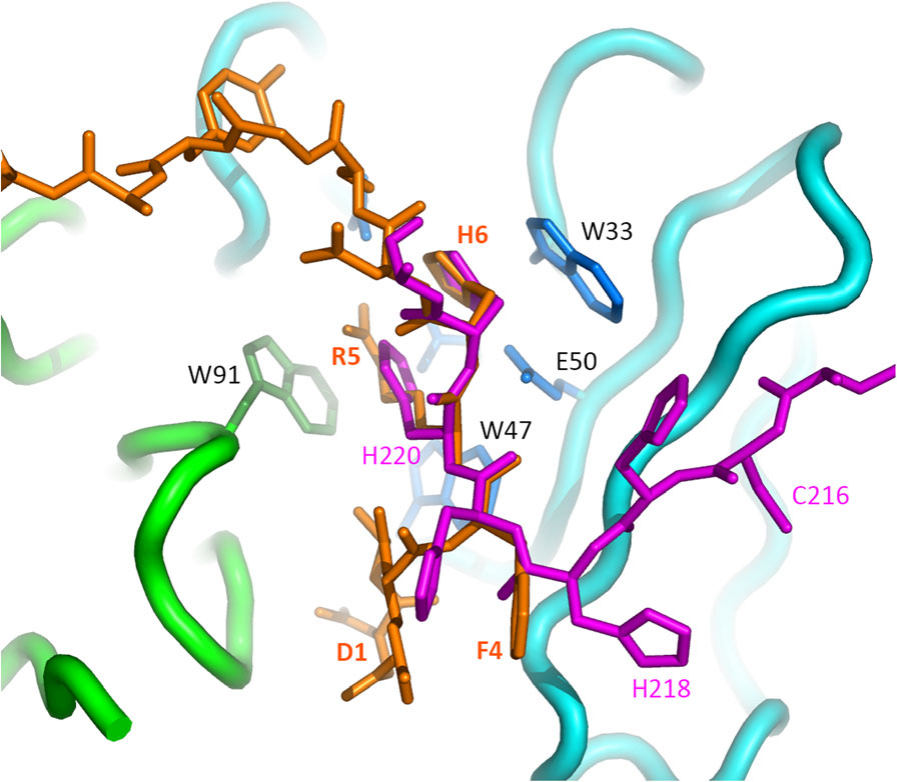
Superposition of the hexahistidine tag bound to the C706 antigen-binding fragment (Fab) (3mcl) on the β-amyloid 1–16 peptide sequence complex. *Green* = variable domain of the light chain (VL), *cyan* = variable domain of the heavy chain (VH), *orange* = Aβ_16_, and *magenta* = His tag

In the crystal structure of C706 determined earlier (3mcl; [13]), the His tag of one Fab occupies the antigen-binding site of another Fab. Two consecutive histidine residues fill the central pocket so that they stack against Trp91(L) and Trp33(H) very much like Arg5 and His6 of Aβ_16_ in the present complex. Superposition of the His tag bound to the C706 Fab on the structure of the complex reveals that the four central residues of the ligands overlap remarkably well (Fig. 2). The root-mean-square deviation (RMSD) for the backbone atoms of these four residues is 0.66 Å. The ability of C706 to bind a polyhistidine sequence prompted us to use a tagless Fab in the present study. The initial attempt to crystallize the C706-Aβ complex yielded crystals that contained only Fab with the His tag occupying the binding site [13].

Comparison of the unbound Fab structure with that in complex with Aβ shows no significant changes in the individual CDR conformation. The only exception is the tilt of CDR H3 by 8 degrees, so that the tip of the CDR loop travels over 2 Å toward Aβ. The VL and VH domains can be superimposed with RMSDs of, respectively, 0.31 Å and 0.38 Å (without CDR H3). Although both domains behave as rigid bodies, their relative orientation changes by 6 degrees, exceeding the normal “breathing” of about 2–3 degrees typical for Fabs [25]. Together with the adjustment of CDR H3, this VL/VH repacking indicates an induced-fit mechanism of Aβ recognition by C706.

**Fig. 3:**
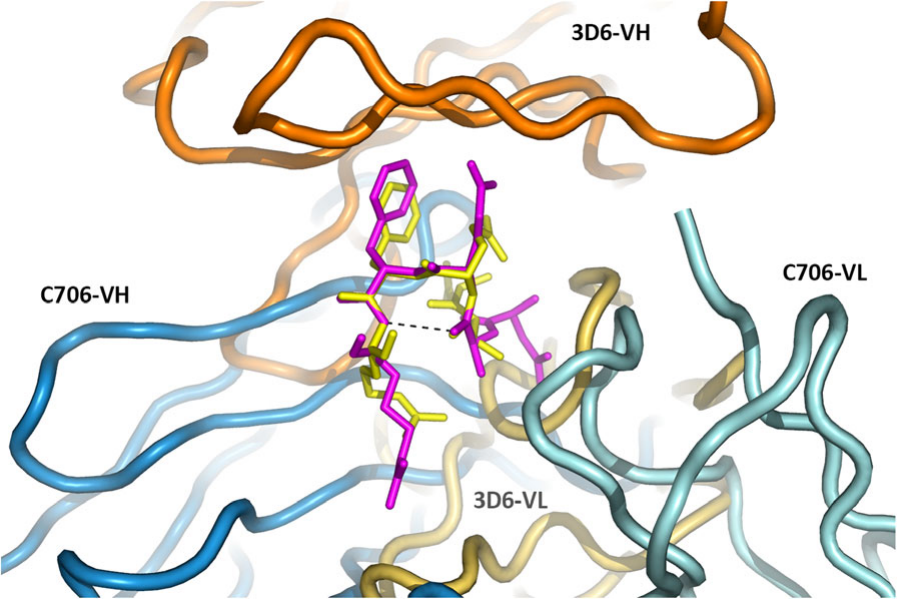
Superposition of β-amyloid (Aβ) residues 1–5 in the C706 complex on the 3D6 complex (1hix). Variable domains of the monoclonal antibodies are shown as tubes (variable domain of the light chain [VL] lighter than variable domain of the heavy chain [VH]), the Aβ peptides as sticks. C706 (*blue*) binds Aβ from the bottom, and 3D6 (*orange*) binds Aβ from the back of the figure. Aβ bound to C706 is shown in *magenta*, and Aβ bound to 3D6 is shown in *yellow*. Hydrogen bond Ala2-Arg5 in Aβ is shown as a *dashed line*

Anti-Aβ mAb 3D6 also recognizes five N-terminal residues of Aβ, although differently from C706. Comparison with the structures of 3D6 (4onf; [26]) and its humanized version bapineuzumab (4hix; [27]) with bound Aβ peptides shows that Aβ residues 2–5 adopt a remarkably similar conformation. Both 3D6 and C706 bind Aβ as a 3_10_ helix stabilized by an H-bond between the Ala2 carbonyl and the amino group of Arg5 (Fig. 3). The Aβ residues 2–5 can be superimposed with an RMSD of only 0.34 Å calculated for all main-chain atoms. Although the conformation of the peptide is virtually identical, the binding mode of the two antibodies is different. In the C706 complex, residues Glu3-Phe4 of Aβ point away from the mAb, whereas in the 3D6 complex, they are immersed in the VL/VH cleft. With respect to the CDRs, the Aβ peptide is rotated by ~ 90° in the two structures.

## Discussion

The crystal structure of the C706-Aβ_16_ complex was determined at high resolution and revealed the antibody-antigen interactions in much detail. Quite unexpectedly, all 16 residues of Aβ_16_ could be traced in the present structure. The Aβ peptide, regardless of its length, is usually disordered beyond the epitope portion in contact with the CDRs. In other words, interactions with antibodies stabilize the Aβ conformation, which otherwise lacks a secondary structure. Whereas C706 binds residues 1–8 of Aβ, the C-terminal half of the peptide is likely stabilized in the crystal through the interactions with a symmetry-related Fab. Although the observed conformation may be affected by crystal contacts, this gives us a unique opportunity to compare the Aβ structure with other structures of this segment available in the Protein Data Bank.

Numerous nuclear magnetic resonance studies demonstrate a wide range of conformations for residues 1–16, asserting monomeric Aβ as a classic example of the intrinsically unstructured protein [28]. The only crystal structure of Aβ covering this segment is that of Aβ_16_ fused to the *Escherichia coli* immunity protein Im7 and stabilized with the WO2 Fab, which binds residues 1–8 (4f37; [29]). The comparison shows that in both structures, ours and theirs, residues 9–16 have no apparent secondary structure. However, residues 9–12 superpose remarkably well, with an RMSD of only 0.3 Å, suggesting some preferred stable conformation.

Interest in antibodies recognizing the N-terminal Aβ segment and specific to both soluble and insoluble forms of Aβ prompted the X-ray studies to establish the link between their structure and in vivo properties. At least eight antibodies have been structurally characterized, providing a detailed view of antibody-antigen interactions (reviewed in [30]). Remarkably, all mAbs except 3D6 bind Aβ in the extended conformation [31–34].

The mode of binding and key interactions are identical in mAbs 10D5, 12A11, 12B4, WO2, and PFA1/2, despite the differences in the sequences and structures. All these mAbs have originated from the same mouse germlines, IGKV1-117 for VL and either IGHV8-8 or IGHV8-12 for VH. The principal recognition element in these mAbs is CDR H2 with the sequence HIWWDDD (in IGHV8-8) or HIYWDDD (in IGHV8-12). The substitution of an aromatic residue at position 52, Tyr for Trp, is well tolerated because it stacks against the aliphatic chain of Arg5 of Aβ. The VL sequences are virtually identical, which ensures the conservation of an important contribution from CDRs L1 and L3. Almost all paratope residues come from CDRs H2, L1, and L3, whereas the most diverse CDR, H3, does not play a significant role in Aβ binding. Therefore, regarding Aβ recognition, the six mAbs are closely related and essentially represent variants of only one antibody.

A very similar extended conformation of Aβ is observed in gantenerumab, a human antibody obtained from a combinatorial phage display library [34]. As in the murine mAbs, the epitope is centered on Phe4, which binds in a deep hydrophobic pocket between VL and VH. Residues 3–6 of Aβ bound to gantenerumab (5csz) and to WO2 (3bkj) can be superimposed with an RMSD of only 0.46 Å. However, gantenerumab exhibits an inverted orientation of Aβ with respect to the CDRs, so that the N-terminus resides at CDR H3 rather than at CDR L3. Whether the extended conformation of Aβ observed in all these antibodies is indicative of a preferred Aβ structure in solution is an open question.

In contrast to those mAbs, 3D6 and C706 bind Aβ in the coiled conformation. In 3D6, residues 1–5 form a regular 3_10_ helix, whereas in C706, the helix is somewhat distorted at Asp1, probably owing to a different CDR environment. The two mAbs have no sequence similarity within the CDRs, because they originated from unrelated mouse germlines, IGKV1-135 and IGHV5-6 for 3D6 and IGKV4-59 and IGHV1-9 for C706. Moreover, the mAbs approach the Aβ peptide from different sides, so the key epitope residues are nonoverlapping (Glu3 and Phe4 in 3D6 versus Arg5 and His6 in C706). Given the differences between 3D6 and C706, it appears particularly interesting that Aβ adopts virtually the same conformation, suggesting that it is one of the stable conformations in the pool of Aβ monomers.

The distinct modes of Aβ recognition observed in the crystal structures prompted us to look into the immunization protocols of these antibodies. All mAbs except gantenerumab were raised in mice; however, the immunogens varied. Aβ_28_ and Aβ_42_ conjugated to the carrier protein were used for 12A11, 12B4, and 10D5 [9, 35]. Aβ_40_ fibrils and CLC-stabilized protofibrils were used for WO2 [36] and PFA1/PFA2 [31], respectively. In all these cases, the outcome was an antibody based on mouse germlines IGKV1-117 and IGHV8-8/12 with a distinct paratope recognizing a unique extended conformation of the N-terminal section of Aβ. Gantenerumab was selected from a combinatorial library by using Aβ_40_ fibrils [34].

3D6 was obtained by immunizing mice with Aβ_7_ conjugated to keyhole limpet hemocyanin [37]. Similarly, Aβ_5_ conjugated to sheep immunoglobulin was used for C706 [13]. In both cases, a short N-terminal Aβ peptide spanning just the epitope residues yielded the antibodies recognizing a coiled conformation of Aβ. One may speculate that in the context of a longer Aβ peptide, such as Aβ_28_ or Aβ_40_, the N-terminal portion tends to adopt an extended conformation, possibly as part of a β-hairpin structure. This may occur in fibrils and protofibrils, as well as in monomeric Aβ preparations, although one can never exclude the presence of oligomers in those samples, given the ease of monomer-oligomer transition [38]. It has been noted that even antibodies recognizing the same extended form of Aβ may be specific to different molecular species [33]. Whether distinct modes of Aβ recognition translate into different pharmacological outcomes remains to be seen.

## Conclusions

Antibody C706 binds residues 1–8 of Aβ, whereas Aβ residues 9–16 that could be traced in the present structure are not in contact with the CDRs. Arg5 and His6 of Aβ occupy the central cleft of the antibody and provide the majority of interactions. Unlike most mAbs, C706 recognizes a coiled rather than extended conformation of Aβ. Comparison with other mAbs targeting the N-terminal section of Aβ suggests that the conformation of the bound peptide may be linked to the immunization protocol and may reflect the preference for the extended conformation in the context of a longer Aβ peptide as opposed to the coiled conformation in the isolated short peptide.

## Abbreviations

Aβ: β-Amyloid
Aβ_16_: β-Amyloid 1–16 peptide sequence
AD: Alzheimer’s disease
APP: Amyloid precursor protein
CDR: Complementarity-determining region
Fab: Antigen-binding fragment
mAb: Monoclonal antibody
RMSD: Root-mean-square deviation
VH: Variable domain of the heavy chain
VL: Variable domain of the light chain
